# Inhibition of Dot1L Histone Methyltransferase Expands Bone Injury-Responsive CXCL12^+^ Stromal Progenitors

**DOI:** 10.64898/2026.04.06.716818

**Published:** 2026-04-09

**Authors:** Marta Stetsiv, Drew Dauphinee, Sakinah Abdulsalam, Shagun Prabhu, Alexander Tress, Kerry Cobb, Archana Sanjay, Rosa M. Guzzo

**Affiliations:** 1 Department of Neuroscience, School of Medicine, UConn Health, 263 Farmington Avenue, Farmington, CT, 06030 USA; 2 Department of Orthopaedic Surgery, School of Medicine, UConn Health, 263 Farmington Avenue, Farmington, CT, 06030 USA; 3 Computational Biology Core, UConn Health, 263 Farmington Avenue, Farmington, CT, 06030 USA

**Keywords:** osteogenic differentiation, Dot1L epigenetic regulation, skeletal stem and progenitors, bone regeneration

## Abstract

The regenerative capacity of adult bone relies on the rapid activation and lineage engagement of skeletal stromal and progenitor cells (SSPCs). While signaling pathways that regulate these processes have been extensively studied, the epigenetic mechanisms that constrain progenitor activation and lineage permissiveness during adult bone repair remain poorly defined. Disruptor of telomeric silencing 1 like (Dot1L), the sole histone methyltransferase responsible for H3K79 methylation, is essential for skeletal development, yet its function in adult skeletal regeneration has not been established. Here, we identify Dot1L as a key epigenetic regulator that limits the early regenerative response to bone injury. Genetic reduction of Dot1L activity in the Prrx1^+^ mesenchymal lineage enhances stromal progenitor activation, proliferative engagement, and differentiation capacity, revealing a previously unrecognized role for Dot1L in restraining progenitor responsiveness in adult bone. Notably, acute pharmacologic inhibition of Dot1L using the selective H3K79 methyltransferase inhibitor EPZ-5676 similarly enhances early progenitor activation, indicating that reduced Dot1L enzymatic activity is sufficient to modulate regenerative engagement. At the cellular level, reduced Dot1L activity expands injury-responsive Cxcl12^+^ stromal populations and increases osteogenic progenitor abundance *in vivo* following injury. Consistent with these cellular changes, Dot1L reduction is associated with accelerated early bone formation *in vivo.* Collectively, these findings position Dot1L as an epigenetic gatekeeper that constrains early progenitor activation during the initial phase of adult skeletal repair.

## INTRODUCTION

The adult bone marrow is composed of a complex stromal compartment that supports homeostatic bone maintenance, remodeling, and bone regeneration. Early foundational studies established that Lepr^+^ perisinusoidal stromal cells represent the principal source of osteoblasts and adipocytes in adult bone marrow, and that these cells remain largely quiescent under homeostatic conditions but become activated following skeletal injury (Zhou et al., 2014b). Closely related Cxcl12-abundant reticular (CAR) cells, located in perisinusoidal niches, are best known for producing niche-supportive factors such as CXCL12 and SCF that regulate hematopoietic stem and progenitor cell maintenance (Ding and Morrison, 2013; Omatsu et al., 2010). Importantly, injury models have revealed that these normally quiescent CXCL12^+^ stromal populations undergo rapid activation, proliferative expansion, and transcriptional reprogramming following bone damage, enabling their migration toward injury sites and differentiation into osteolineage cells (Matsushita et al., 2020). Together, these findings establish perisinusoidal CXCL12^+^ stromal cells as a major injury-responsive skeletal stromal and progenitor cell (SSPC) population in adult bone marrow that contributes directly to regenerative bone formation following injury. Significantly, these stromal populations retain multilineage potential, and effective skeletal repair depends not only on their activation, but also on appropriate regulation of lineage engagement during regeneration.

With advances in experimental approaches integrating single cell-RNA sequencing (scRNA-seq), lineage tracing, and spatial transcriptomics, it is now increasingly clear that bone marrow stromal populations are highly heterogeneous, composed of transcriptionally distinct subsets with divergent lineage biases, proliferative capacities, niche locations, transitional states, and responses to environmental cues (Baccin et al., 2020; Tikhonova et al., 2019). Consistent with this emerging framework, Liao et al. (2025) identified a distinct Adipoq^+^ Osx^+^ endosteal progenitor population in adult bone marrow that contributes to both osteogenic and adipogenic lineages (Liao et al., 2025). These findings demonstrate that osteogenic support within the marrow arises from multiple specialized progenitor subsets with distinct activation states, and lineage biases, raising the question of how such heterogeneous translational progenitor programs are coordinated and regulated during skeletal repair.

Reparative bone formation depends on the ability of normally quiescent SSPCs to activate, proliferate, and differentiate into bone forming osteoblasts. While signaling pathways governing SSPC activation and lineage commitment have been extensively studied, including Wnt, BMP, and Notch signaling pathways (Salhotra et al., 2020), the epigenetic mechanisms that determine the timing and magnitude of progenitor activation during adult skeletal repair remain poorly understood. Because adult stromal progenitors retain the capacity to engage multiple lineage programs in defined culture conditions, assessment of intrinsic lineage potential provides a useful framework for interrogating progenitor regulation without presuming equivalent lineage outcomes *in vivo*. It remains unclear whether epigenetic regulators primarily enforce lineage restriction or gate progenitor activation (Stetsiv et al., 2025a). Consistent with emerging models, epigenetic regulators may act in a context dependent manner to modulate progenitor quiescence, activation, or lineage permissiveness within specific stromal subsets, rather than functioning as global regulators across all skeletal stromal and progenitor populations.

Disruptor of Telomeric Silencing 1 Like (Dot1L) is the sole histone H3K79 methyltransferase (H3K79me1/2/3) (Nguyen and Zhang, 2011) and a dosage sensitive regulator of progenitor behavior during development. Dot1L is required for proper proliferation, lineage commitment, and organogenesis across multiple tissues, including cardiac, neural, spinal cord, and hematopoietic lineages (Feng et al., 2010; Ferrari et al., 2020; Gray de Cristoforis et al., 2020; Pursani et al., 2018; Roidl et al., 2016). In neural stem and progenitor populations, Dot1L inhibition promotes increased neurogenic differentiation (Appiah et al., 2023). In the skeletal system we have shown that developmental deletion of Dot1L in Prrx1^+^ limb mesenchyme results in profound skeletal dysplasia, including long bone shortening, disrupted growth plate architecture, and reduced trabecular bone, even under haploinsufficient conditions (Sutter et al., 2021).

In contrast to its essential role in promoting progenitor proliferation and lineage progression during development, emerging evidence indicates that Dot1L plays a distinct role in adult tissues by maintaining cell identity and restraining inappropriate lineage activation. Conditional loss of Dot1L in mature T and B lymphocytes leads to aberrant lineage programs and dysregulated effector responses (Aslam et al., 2021; Scheer et al., 2020). In skeletal tissues, Dot1L downregulation has been linked to enhanced periosteal-driven mandibular bone regeneration, whereas Dot1L overexpression suppresses osteogenic differentiation (Jiang et al., 2025).

Collectively, these findings suggest that Dot1L promotes lineage progression during development, but functions in post-developmental contexts as an epigenetic brake that preserves progenitor quiescence or identity likely by limiting the breadth and timing of lineage priming rather than enforcing a single differentiation outcome. Given the diverse sources of skeletal progenitors involved in bone maintenance and repair, this framework raises the question of whether Dot1L similarly regulates the activation and lineage engagement of SSPCs within the adult bone marrow.

In this study, lineage priming is inferred through a combination of intrinsic differentiation assays and transcriptional profiling of injury-responsive stromal populations. Because skeletal repair requires rapid activation of normally quiescent marrow SSPCs we sought to determine whether Dot1L functions to constrain early progenitor expansion and regulate lineage priming during adult bone repair. By interrogating both cell intrinsic progenitor behaviors in vitro and regenerative responses *in vivo*, this study tests how genetic and pharmacologic reduction of Dot1L activity influences stromal progenitor behavior, injury responsive populations, and early regenerative bone formation. Using an established model of bone marrow injury that induces rapid intramembranous bone formation, together with single cell transcriptomic profiling and quantitative analyses of regenerative bone formation, we define how Dot1L shapes marrow stromal cell responses during the early phases of skeletal repair.

## MATERIALS AND METHODS

### Mice

All other breeding and genotyping strategies were followed as previously published (Sutter et al., 2021). C57BL/6 (000664), and Prrx1Cre (005584) were obtained from The Jackson Laboratory. Female Dot1L^fl/fl^ originally generated and described by Bernt et al. (Bernt and Armstrong, 2011) were crossed with Prrx1Cre male mice as we have previously described (Sutter et al., 2021) to generate Dot1L^fl/wt^:Prrx1 mice. Dot1L^fl/wt^:Prrx1Cre male offspring were mated with Dot1L^fl/fl^ females to produce conditional knockout mice (Dot1L^fl/fl^:Prrx1Cre, designated as fl/fl:Prrx1), conditional heterozygous or haploinsufficient mice (Dot1L^fl/wt^:Prrx1Cre, designated as fl/wt:Prrx1) and wild-type littermate controls which did not express Cre (Dot1L^fl/fl^ or Dot1L^fl/wt^). Female and male mice were used for all experiments.

All mice were on the C57BL/6 background, and sex- and age-matched mice were used for experiments. Mice were housed on a 12-hour light-dark cycle with ad libitum access to food (Teklad Global 19% Protein Extruded Diet, #2918) and water under specific pathogen-free conditions at UConn Health Center for Comparative Medicine. Animal procedures were conducted in accordance with protocols approved by the Institutional Animal Care and Use Committee (IACUC) at UConn Health.

### Isolation of bone marrow stromal cell and *in vitro* proliferation assays

Total bone marrow was harvested from the hindlimbs of adult (16–24-week-old) Dot1L^fl/fl^ and Dot1L^fl/fl^:Prrx1Cre mice. Marrow contents were flushed with sterile phosphate-buffered saline (PBS) using a 25-gauge needle and filtered through a 70 μm nylon strainer. Cells were counted using an automated cell counter (Bio-Rad TC20) and cultured in growth medium consisting of DMEM supplemented with 10% fetal bovine serum and 1% penicillin–streptomycin. Cultures were maintained at 37 °C in a humidified incubator with 5% CO_2_.

For colony-forming unit (CFU) assays, freshly isolated bone marrow cells from each genotype were plated at 2.12 × 10^6^ cells per well in 6-well plates and cultured for 10–11 days. Cells were fixed in 10% formalin and stained with alkaline phosphatase (ALP) solution to assess osteoblastic colony formation (CFU-Ob). Counter-staining with hematoxylin was used to visualize total fibroblastic colony formation (CFU-F). Plates were imaged using a flatbed scanner.

Cell proliferation assays were performed using the Cell Counting Kit-8 (CCK-8; Sigma-Aldrich). To enrich for adherent stromal cells and allow recovery from isolation-related stress, bone marrow stromal cells from each genotype were passaged once prior to the assay. Cells were then seeded at densities of 7.5 × 10^3^ or 12 × 10^3^ cells per well in 96-well plates and cultured for 5 days. CCK-8 reagent was added directly to the culture medium and incubated for 4.5 hours, after which absorbance was measured at 450 nm using a microplate reader (PerkinElmer EnSpire 2300). All values were background-corrected and normalized to the mean of the control group.

### Bone Marrow Stromal Cell Differentiation

Osteogenic differentiation was induced using passage 1 bone marrow stromal cells (BMSCs) seeded at 2.2 × 10^5^ cells per well in gelatin-coated 12-well plates. Upon reaching confluence, cultures were exposed to osteogenic differentiation medium consisting of growth medium supplemented with 50 μg/mL ascorbic acid and 8 μM β-glycerophosphate. Mineralized matrix deposition was assessed on days 10 and 14 of differentiation by 2% Alizarin Red staining of formalin-fixed cultures. Plates were air-dried, imaged using a flatbed scanner and Alizarin Red staining was quantified by solubilization in 10% acetic acid followed by heating at 85 °C for 10 minutes. Neutralized supernatants (10% ammonium hydroxide, pH 4.1–4.5) were analyzed by absorbance at 405 nm using a microplate reader (PerkinElmer EnSpire 2300).

Adipogenic differentiation assays used passage 1 BMSCs seeded at a density of 2.5 × 10^5^ cells per well in 12-well plates. Cells were cultured until approximately 80% confluency prior to adipogenic induction. Adipogenesis was induced as previously described in growth media supplemented with 3 μM rosiglitazone, 1X insulin–transferrin–selenium (ITS), and 1 μM dexamethasone (Heimann et al., 2023). Media was replaced every 2 days. Accumulation of intracellular lipid droplets was assessed on day 5 of differentiation by Oil Red O staining of formalin-fixed cells. Oil Red O staining was visualized by brightfield microscopy.

### Quantitative PCR analysis

BMSCs undergoing osteogenic differentiation were washed with phosphate-buffered saline (PBS) and lysed directly in TRIzol reagent (Thermo Fisher Scientific) by scraping. Total RNA was isolated according to the manufacturer’s standard protocol, including phase separation with chloroform, RNA precipitation with isopropanol, and ethanol washes. RNA pellets were resuspended in nuclease-free water and treated with DNase I to remove genomic DNA contamination. cDNA was synthesized from purified RNA using iScript Reverse Transcription Supermix (Bio-Rad) according to the manufacturer’s instructions. The resulting cDNA was diluted to a final concentration of 5 ng/μL and used as input for quantitative PCR. qPCR was performed using Advanced Universal SYBR Green Supermix (Bio-Rad) on a CFX96 Touch Real-Time PCR Detection System (Bio-Rad). Reactions were run under standard cycling conditions with melt curve analysis to confirm product specificity. Relative mRNA expression levels were calculated using the comparative cycle threshold (Ct) method (ΔΔCt), normalized to β-actin (*Actb*) as the internal control. Gene expression values are presented as 2^−ΔΔCt^. Murine oligonucleotide primer sequences are provided in Table1.

### Mechanical Bone Marrow Injury

Bone marrow injury was performed as we have previously described (Stetsiv et al., 2025b). Surgeries were performed on the left femur of anesthetized female Dot1L^fl/wt^:Prrx1Cre and Cre negative (Dot1L^fl/wt^ or Dot1L^fl/fl^) controls aged 10–12 weeks. Surgeries were performed under aseptic conditions. The distal femur was accessed through the intercondylar notch, the medullary canal reamed and flushed with sterile saline until clear. The contralateral femur served as uninjured controls. Mice received extended-release buprenorphine for analgesia (Ethiqa XR, 3.25 mg/kg), and were monitored post-operatively for recovery and general health. For studies of bone formation, mice were injected with calcein mineral label on days five and six post-surgery and mice were euthanized seven days post-surgery for tissue harvest.

In a separate cohort, wild-type C57BL/6 mice were treated with the selective Dot1L inhibitor EPZ-5676 (Eurofins Advinus) to achieve systemic inhibition of DOT1L methyltransferase activity. EPZ-5676 was prepared in 5% DMSO diluted in corn oil and administered via intraperitoneal injection at a dose of 35 ug/g of body weight per injection (Malcom et al., 2021). Mice received twice-daily dosing for 4 days prior to bone marrow injury, followed by once-daily dosing for 3 days post-injury until tissue harvest.

### *In Vivo* EdU labeling and flow cytometric analysis

Dot1L^fl/fl^:Prrx1Cre and Prrx1-Cre 6-months-old male mice received a single intraperitoneal injection of 5-ethyl-2’-deoxyuridine (EdU) at 50 μg/g of body weight and maintained a 4-hour EdU labeling period to ensure adequate incorporation prior to harvest. Following euthanasia, one femur and one tibia were collected from each mouse, and the epiphyses were removed. Bone marrow was flushed with sterile PBS using a 25-gauge needle, and cell suspensions from both bones were pooled per animal. The marrow was filtered through a 70 μm nylon mesh (Nytex) to obtain a single-cell suspension. Red blood cells were lysed with ACK buffer during the permeabilization step of the Click-iT reaction. Total cell numbers were obtained using a Bio-Rad automated cell counter, and cells were aliquoted to ensure consistent staining across conditions. For each experimental sample, 10 million cells were allocated for staining, and 2–4 million cells were processed for the fluorescence-minus-one (FMO) controls, single stain, and unstained controls used for gating.

Cells were incubated with an antibody cocktail (CD45-FITC, CD31-FITC, and Ter119-FITC;1:200; Table 2) for 30 minutes on ice in the dark followed by washes in PBS containing 1% bovine serum albumin (BSA). Surface-labeled cells were fixed and permeabilized using the Click-iT EdU Alexa Fluor 647 Flow Cytometry Kit (Thermo Fisher) according to the manufacturer’s protocol. After two washes in the Click-iT kit buffer, fixed cells were stored overnight at 4° C in the dark. The following day, EdU incorporation was detected using Alexa Fluor 647 azide and nuclear DNA was counter stained with Hoechst 33342 (1:2000 dilution) for 30 minutes at room temperature in the dark.

Flow cytometric analysis was performed on a BD FACSymphony A5 SE analyzer (BD Biosciences). Compensation was calculated using single-stained controls and gating strategies were defined using Fluorescence Minus One (FMO) and unstained controls. Samples were acquired at low speed to minimize coincident events and at least 100,000 Lineage^−^ events were collected per sample. Post-acquisition analysis was performed using FlowJo v10.10.0. Debris and doublets were excluded using standard forward and side scatter parameters and FSC-A/FSC-H gating. Lineage^−^ cells were identified as CD45^−^CD31^−^Ter119^−^. EdU incorporation was quantified within the Lineage^−^ compartment. Gating hierarchies were established using FMO, single-stain, and unstained controls to ensure specificity of EdU-AF647 and tdTomato signals. Flow cytometry data were analyzed and quantified using FlowJo software (version 10.10.0, BD Biosciences).

### Microcomputed Tomography (Micro-CT)

Femurs were fixed in 10% formalin, rinsed in PBS, and stored in 70% ethanol prior to scanning. Structural analysis of day 7 post-injury femurs was performed using cone-beam x-ray micro-computed tomography (μCT40, Scanco Medical AG) with an isotropic voxel size of 8 μm (55 kVp, 145 μA, 500 ms integration). Three-dimensional reconstruction and morphometric analyses were performed using Scanco Evaluation software (v7.0). The region of interest (ROI) encompassed the intramedullary cavity of the distal femur corresponding to the ablated site. A 600-slice span capturing the injured area below the growth plate was contoured manually on every 10^th^ slice to ensure consistent sampling across animals. Mineralized tissue was segmented using a global threshold (260 mg HA/cm^3^) applied consistently across all samples. Standard morphometric parameters, including bone volume fraction (BV/TV), trabecular number (Tb.N), and trabecular thickness (Tb.Th) were calculated using 3D direct methods.

### Histology and Histochemical Staining

To label newly mineralizing bone, mice were injected with calcein (i.p, 20 ug/g body weight, Sigma-Aldrich) 24 hours before harvesting. Following micro-CT imaging, femurs were processed for frozen histology to preserve fluorophore integrity. Bones were briefly rinsed in PBS, cryoprotected in 30% sucrose in PBS overnight at 4 °C, embedded in optimal cutting temperature (OCT) compound (Tissue-Tek) and frozen on dry ice. Sections (8 μm) were mounted on cryo-adhesive tape (Cryofilm 3C, Kawamoto). Calcein fluorescence with DAPI counterstain, was imaged using a Nikon Eclipse 50i or ZEISS Axioscan. To assess mineralized bone matrix, adjacent sections were stained with von Kossa by immersing in 5% silver nitrate and UV crosslinking (120 mJ/cm^2^, twice). Brightfield images were captured using an Olympus IX71 microscope.

### Immunofluorescence staining

Immunofluorescence staining was performed on frozen bone sections collected 7 days post-injury. For Osterix staining, sections were permeabilized in 0.1% Tween-20 in PBS for 15 min, followed by blocking in 10% normal goat serum (NGS) and 2% BSA in PBS for 1h at RT. Sections were incubated overnight at 4°C with anti-Osterix primary antibody (1:500; Abcam ab209484) diluted in blocking solution. After washing in PBS containing 0.1% Tween-20, sections were incubated with Alexa Fluor 647-conjugated goat anti-rabbit secondary antibody (1:300; Thermo Fisher, A21244) for 1h at RT, followed by additional washes prior to mounting. For Cxcl12 staining, sections were rehydrated and post-fixed in 4% paraformaldehyde, followed by antigen retrieval using Proteinase K (5 μg/mL in 50 mM Tris-HCl, pH 7.5–8.0) for 5 min. Sections were permeabilized in 0.05% Triton X-100 and blocked in 10% NGS, 2% BSA, and 0.05% Triton X-100. Primary antibody incubation (anti-Cxcl12, 1:100; Thermo Fisher Sci, PA589116) was performed overnight at 4 °C in antibody dilution buffer (PBS supplemented with 5% NGS, 1% BSA, and 0.025% Triton X-100). Following washes in PBS containing 0.05% Tween-20, sections were incubated with Alexa Fluor 647-conjugated goat anti-rabbit secondary antibody (1:300) for 1 h at room temperature. Sections were washed and mounted with DAPI-containing glycerol mounting medium. Images were obtained using Zeiss Imager.Z1 or Zeiss Axioscan.

### Single-Cell RNA Sequencing

Bone marrow cells were isolated from injured femurs at 4 days post-surgery. Cells from 4–5 mice of both sexes per genotype were pooled for a single sample for each genotype. For scRNA sequencing, cells were fixed overnight following the 10X Genomics GEM-X Flex protocol (10X). Cells were stained and sorted for CD45^−^, CD31^−^, and Ter119^−^populations using a FACS Aria II (BD) (detailed description in Supplemental Figure 4). Library preparation and sequencing were performed by the Single Cell Biology Laboratory (SCBL) at The Jackson Laboratory for Genomic Medicine using 10X Chromium platform (5’ Gene Expression) using 5,000–10,000 cells/sample with a target depth of 365K-387K reads/cell. The GEM-X Flex-seq probe-based approach utilized the Chromium Mouse Transcriptome Probe Set v1.1.1 targeting 19,070 genes. Illumina base call (BCL) files were converted to FASTQs using bcl2fastq v2.20.0.422 (Illumina) and trimmed to a 28-10-10-90 asymmetric read configuration. The resulting FASTQ files were processed using the Cell Ranger multi pipeline (10x Genomics, v9.0.1). Reads were aligned to the GRCm39 probe set reference, and the probe barcodes were used to demultiplex the pooled samples into individual datasets. Filtered feature barcode matrix files were used for downstream clustering and analysis using Scanpy v1.11.4. To remove uninformative genes, we removed genes present in fewer than 10 cells. Low quality cells were excluded if they expressed fewer than 200 genes or deviated by more than 5 median absolution deviations for metrics including gene counts, total counts, proportion of top 20 expressed genes, and mitochondrial content (Supplemental Figure 5). Ambient RNA was removed using SoupX (v1.6.2), and doublets were identified using scDblFinder (v1.23.4). Batch correction was performed using HarmonyPy (v0.0.10). Data were transformed using the shifted logarithm for clustering and differential expression analysis (log2fold change). Cells were clustered with the Leiden algorithm based on the top 4,000 variable genes (Seurat v3 method), using 20 principal components neighbor computation. Cell clusters were annotated by marker genes and log2 fold-change differences between genotypes were calculated in Scanpy.

### Statistical Analyses

Statistical analyses were performed using GraphPad Prism (Version 10.5.0). All quantitative data are presented as mean ± standard deviation (SD), unless otherwise indicated. Statistical comparisons between two groups were performed using Student’s *t*-tests. Comparisons involving multiple groups were analyzed by one-way or two-way analysis of variance (ANOVA), followed by Tukey’s post hoc multiple-comparison tests, as appropriate. For scRNA-seq analyses, differential gene expression was assessed by Wilcoxon ranking, using log-transformed data and appropriate statistical thresholds as described in the Methods.

## RESULTS

### Dot1L is broadly expressed across Cxcl12^+^ marrow stromal populations.

To determine whether the chromatin modifier Dot1L is positioned to regulate adult bone marrow stromal progenitor populations, we first examined its expression within a compiled scRNA-seq dataset integrating murine bone marrow cells profiled across multiple experimental and physiological conditions (Dolgalev and Tikhonova, 2021). This harmonized dataset encompasses diverse marrow cell types, including stromal, hematopoietic, endothelial, and other niche-associated populations, and is organized into transcriptionally defined clusters annotated based on established lineage and niche marker expression (Supplemental Figure 1A). Projection of Dot1L expression onto this clustered dataset revealed broad transcript detection across marrow populations, with consistent expression within multiple stromal clusters (Supplemental Figure 1B). To further contextualize Dot1L expression, we generated dot plots comparing the relative expression level and the fraction of cells expressing Dot1L alongside additional histone-modifying enzymes and chromatin-associated regulators across stromal clusters (Supplemental Figure 1C). This analysis demonstrates that Dot1L is expressed in parallel with other epigenetic regulators implicated in progenitor maintenance and lineage regulation (Stetsiv et al., 2025a), situating Dot1L within a broader chromatin-regulatory landscape active in bone marrow stromal populations.

**Figure 1.**
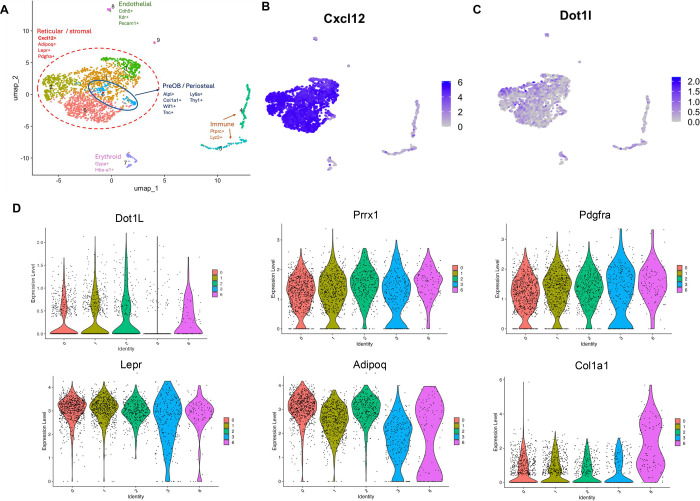
*Dot1L* expression within subsets of Cxcl12^+^ bone marrow stromal cells. (**A**) UMAP of unsupervised clustering revealed transcriptionally distinct sub-populations of Cxcl12+ Lineage(−) mouse bone marrow cells (GSE136979;(Matsushita et al., 2020)). The population was largely defined by reticular stromal properties including the expressions of *Cxcl12*, *Adipoq*, *Lepr*, and *Pdgfra* (dashed red outline). A subset of stromal cells displayed a transcriptional profile consistent with pre-osteoblast/periosteal lineage identity (blue outline). Additional clusters correspond to endothelial, immune, and erythroid populations. UMAP feature plots using color to indicate gene expression (log CPM) of *Dot1L* (**B**) and *Cxcl12*, *Lepr, Pdgfra* and *Prrx1* (**C**).

To more precisely define Dot1L expression within stromal populations most relevant to injury repair, we analyzed a previously published Cxcl12-enriched single-cell RNA-sequencing dataset generated from bone marrow stromal cells isolated from uninjured murine femurs (GSE136979; (Matsushita et al., 2020)). Unsupervised clustering revealed transcriptionally heterogeneous populations within the Cxcl12+ marrow niche, including largely reticular and stromal populations marked by canonical niche-associated genes such as *Adipoq, Lepr,* and *Pdgfra* (Figure 1 A, B, D). Within this broader stromal compartment, a smaller subset of this population was identified as pre-osteoblast/periosteal-like, marked by expression of *Alpl, Col1a1, Wif1, Tnc, Ly6a,* and *Thy1* (Figure 1A). Additional non-stromal clusters corresponding to endothelial, immune, and erythroid populations were also identified. *Dot1L* transcripts were broadly detected throughout the reticular and stromal populations within this marrow microenvironment (Figure 1C). Overlay of stromal lineage marker expression further confirmed overlapping expression profiles of *Dot1L* with *Cxcl12+* subset expressing *Lepr, Pdgfra,* and *Prrx1* (Figure 1D), indicating that Dot1L is expressed across marrow-resident mesenchymal and stromal progenitor compartments relevant to skeletal repair. Consistent with emerging models emphasizing chromatin regulators as integrators of progenitor quiescence, activation, and injury-induced fate transitions in bone (Stetsiv et al., 2025a), additional feature plots of epigenetic regulators implicated in osteogenic regulation showed that Dot1L is part of a wider chromatin-regulatory landscape active within the Cxcl12^+^ marrow niche (Supplemental Figure 1D). Given its unique role as the sole H3K79 methyltransferase, these findings formed the rationale for the present study, which tested whether Dot1L functions as a non-redundant epigenetic regulator of adult bone marrow SSPC proliferation, fate transitions, and injury-induced osteogenesis.

### Dot1L deletion in Prrx1^+^ bone marrow stromal cells enhanced cellular expansion and osteogenic colony formation.

**Figure 2.**
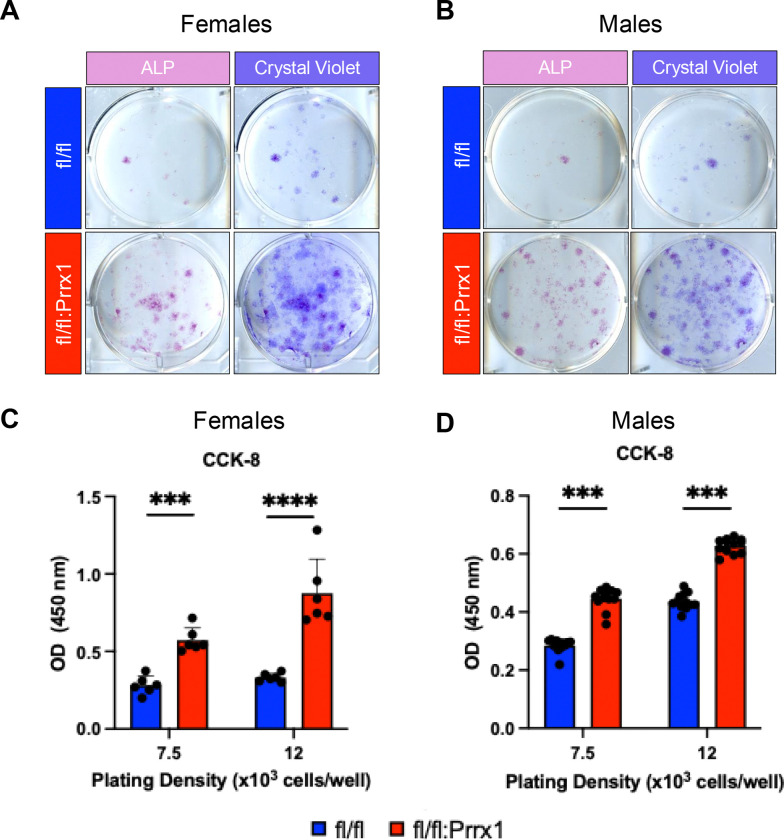
*Dot1L* deletion by Prrx1Cre increased bone marrow colony forming units. (**A, B**) Representative images of crystal violet (CV) staining and alkaline phosphatase activity (ALP) of colony forming units from bone marrow harvested from adult female and male Dot1L^fl/fl^ and Dot1L^fl/fl^:Prrx1 mice. (**C,D**) Quantitative cell proliferation assay using the CCK-8 kit was performed in BMSCs from Dot1L^fl/fl^ and Dot1L^fl/fl^:Prrx1Cre mice from both sexes. Cells (passage 1) were plated at two different plating densities (7.5 × 10^3^ and 12 × 10^3^ cells per well of 96 well plate). Background corrected absorbance measurements at 450nm is shown for cells from each genotype and cell seeding density. Each assay consisted of a minimum of 3 technical replicates per condition per assay, repeated at least 3 times. Graphs presented as mean ± SD; *** *p* ≤ 0.001; **** *p* ≤ 0.0001.

To test the functional contribution of Dot1L in adult skeletal progenitors, we initially examined the colony forming units within BMSCs harvested from Dot1L^fl/fl^:Prrx1Cre and control mice (Dot1f^l/fl^). BMSCs from Dot1L^fl/fl^:Prrx1Cre mice yielded more alkaline phosphatase-positive (ALP^+^) colonies, indicative of an enhanced osteogenic potential, as well as a dramatic increase in CFUs shown by crystal violet staining (Figure 2A, C). To further evaluate changes in proliferative capacity attributed to loss of Dot1L function, we performed colorimetric cell counting kit-8 (CCK-8) assays at two plating densities. BMSCs from Dot1L^fl/fl^:Prrx1Cre mice exhibited a marked and highly significant increase in absorbance as compared to BMSCs from Dot1L^fl/fl^ controls, reflecting a robust enhancement of proliferative activity. Specifically, absorbance increased by 2.0-fold in female-derived cells and by 1.7-fold in male-derived cells (*p* ≤ 0.001; Figures 2B, D). This proliferative advantage was robust and reproducible across biological replicates and repeat experiments. Importantly, the magnitude of this response was comparable between sexes, indicating that Dot1L regulates BMSC expansion in a sex-independent manner. Together, these *in vitro* assays show that Dot1L depletion enhances both clonal output and proliferative capacity of adult marrow stromal progenitors.

### Dot1L loss in the Prrx1 lineage promotes bone marrow stromal progenitor expansion and cell cycle entry in vivo

**Figure 3.**
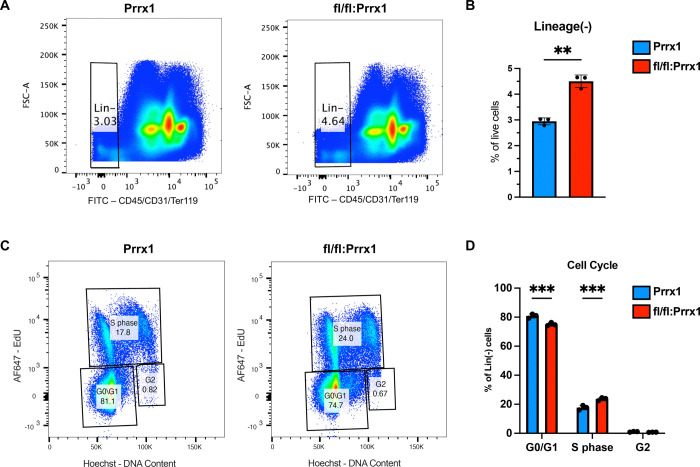
*In vivo* EdU labeling confirms increased proliferative capacity of Lineage^negative^ bone marrow cells in Dot1L^fl/fl^:Prrx1Cre mice. (**A**) Pseudocolor dotplot depicting distribution of CD45^−^CD31^−^Ter119^−^ (Lineage^−^) cells in bone marrow-derived cells from Prrx1-Cre and Dot1L^fl/fl^:Prrx1 mice. **(B)** Quantitative analysis of the relative fractions of Lineage^negative^ populations in Dot1L^fl/fl^:Prrx1 mice as compared to Prrx1Cre only controls. Representative plots show DNA content by Hoechst versus EdU incorporation to distinguish cell-cycle phases within the Lineage^−^ stromal and progenitor population from Prrx1-Cre controls (left) and Dot1L^fl/fl^:Prrx1 mice (right). Cells in G0/G1 were identified as Hoechst low and EdU negative, S phase cells as EdU^+^, and G2/M cells as Hoechst high and EdU negative. (**D**) Quantification of G0/G1, S phase, and G2 cell-cycle distribution in the Lineage^−^ population. Data presented as mean ± SD; * *p* ≤ 0.05 ** *p* ≤ 0.01

Considering the increased proliferative and colony-forming potential in Dot1L-deficient BMSCs, we examined whether the Prrx1Cre-driven loss of Dot1L also causes measurable changes in bone marrow stromal progenitor pool size *in vivo*. Adult Dot1L^fl/fl^:Prrx1Cre and Prr1Cre mice were pulsed with EdU and bone marrow cells were harvested directly and analyzed by flow cytometry (gating strategy in Supplemental Figure 2) without intervening culture, allowing assessment of proliferation and progenitor abundance under physiological conditions. Flow cytometric profiling revealed a significantly increased proportion of CD45^−^CD31^−^Ter119^−^ (Lineage^−^) cells in the bone marrow of Dot1L^fl/fl^:Prrx1 mice (4.5% ± 0.25) compared with wild-type Prrx1Cre (2.95% ± 0.14) controls (*p* = 0.002) (Figures 3A, B). Because CD45^−^ cells represent a heterogeneous compartment enriched for nonhematopoietic stromal populations including Prrx1 lineage cells and early osteolineage-biased progenitors, an increase in this fraction may reflect expansion of stromal progenitors or altered proliferative dynamics.

To assess whether this expanded progenitor pool exhibits increased *in vivo* proliferative activity, we quantified EdU incorporation within the Lineage^−^ fraction. Cell-cycle analysis through combined DNA content by Hoechst and EdU incorporation measurements showed a redistribution of cells from G0/G1 into S phase, whereas the G2/M fraction was not markedly altered, consistent with enhanced cell-cycle entry (Figures 3C, D). The positive shift in cells entering S-phase (23.63% ± 1.1 in Dot1L^fl/fl^:Prrx1Cre vs 17.7% ± 1.6 in control) was accompanied by a corresponding decrease in G0/G1 from 80.8% ± 1.42 in the controls to 75.1% ± 1.1 in Dot1L^fl/fl^:Prrx1Cre mice. The G2 fraction did not significantly change between the two groups.. Together with the enhanced clonogenic capacity and proliferative output observed *in vitro*, these *in vivo* data indicate that loss of Dot1L in the Prrx1 lineage promotes expansion of the marrow stromal progenitor compartment through increased cell-cycle entry.

### Dot1L deletion drives increased osteogenic and adipogenic differentiation in cultured BMSCs.

**Figure 4.**
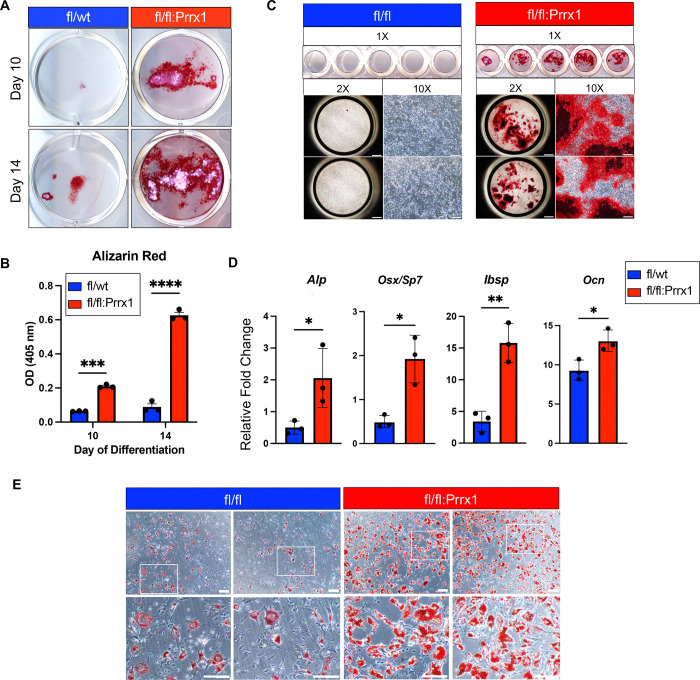
Prrx1-driven loss of Dot1L expression increased osteogenic and adipogenic differentiation capacity in bone marrow stromal cells. (**A**) Representative Alizarin Red staining of mineral deposits in osteogenic BMSCs cultures from male Dot1L^fl/wt^ control and Dot1L^fl/fl^:Prrx1 mice. Cultures underwent osteogenic differentiation at passage 1. Cultures from each condition were stained on days 10 and 14 of osteogenic differentiation. (**B**) Quantitative analysis of Alizarin Red stain by solubilization and absorbance at 405 nm. (**C**) Scanned images and representative high magnification images (2x, 10x) of Alizarin Red stain depicting mineral deposition in Dot1L^fl/fl^ control cells (left) vs Dot1L^fl/fl^:Prrx1 (right) at day 12 of osteogenic differentiation using passage 3 cells. (**D**) mRNA expression of early (*Alp*), intermediate (*Osx/Sp7, Ibsp*), and late (*Ocn*) osteogenic markers on day 7 of differentiation. mRNA expression presented as relative fold change normalized to β-actin expression. Graphs presented as mean ± SD; * *p* ≤ 0.05 ** *p* ≤ 0.01 *** *p* ≤ 0.001; **** *p* ≤ 0.0001 (**E**) Representative Oil Red O staining of lipid droplets formed within 5 days of adipogenesis in BMSCs from male Dot1L^fl/wt^ control and Dot1L^fl/fl^:Prrx1 mice. Scale bar, 100um.

We next asked whether the enhanced stromal progenitor cell expansion observed in Dot1L-deficient BMSCs was accompanied by a heightened osteogenic differentiation capacity. BMSCs from Dot1L^fl/fl^ and Dot1L^fl/fl^:Prrx1Cre mice were differentiated toward the osteoblast lineage *in vitro* and stained with Alizarin red. Alizarin red staining of cultures following 10 and 14 days of differentiation showed increased early mineralizing activity in cultures from Dot1L^fl/fl^:Prrx1Cre mice relative to controls (Figure 4A). Quantitative analysis of Alizarin red staining revealed a significant increase in mineral deposition in Dot1L-deficient cultures at both time points. We detected a 3-fold increase at day 10 and a 7-fold increase on day 14 when comparing Alizarin Red absorbance readings between Dot1L^fl/fl^:Prrx1Cre and Dot1L^fl/fl^ control cells (Figure 4B). Notably, even after three consecutive passages, conditions that usually diminish osteogenic potential of primary BMSCs, Dot1L-deficient cells continued to exhibit markedly increased Alizarin Red mineral staining compared to control cells (Figure 4C). The persistence of this phenotype across serial passaging indicates that Dot1L loss enforces a sustained, cell-intrinsic osteogenic bias rather than a transient differentiation advantage. Consistent with increased mineralization, quantitative analyses of the mRNA expressions of canonical osteoblast genic markers at day 7 of differentiation revealed a 4-fold increase in *Alp* and *Osx/Sp7, a* 5.5-fold increase *Ibsp*, and a 1.5-fold difference in *Ocn* in Dot1L conditional knockout versus vs control cells (Figure 4D). These observations indicate that loss of Dot1L in BMSC leads to enhanced osteogenic commitment and mineralized matrix production.

In parallel, we assessed adipogenic differentiation to determine whether Dot1L loss broadly alters mesenchymal lineage commitment. Under adipogenic induction conditions, Dot1L-deficient BMSCs exhibited enhanced adipocyte formation compared to controls, as evidenced by increased lipid accumulation following Oil Red O staining (Figure 4E). These findings indicate that Dot1L deletion enhances adipogenic differentiation capacity alongside osteogenesis. Together, these results demonstrate that loss of Dot1L increases the multilineage differentiation potential of adult BMSCs, promoting both osteogenic and adipogenic outcomes. This phenotype is consistent with a model in which Dot1L normally restrains mesenchymal progenitor activation and lineage priming, rather than selectively enforcing commitment to a single differentiation pathway.

### Single-Cell RNA sequencing showed increased expansion of osteolineage cells during bone marrow regeneration under conditions of genetic and pharmacologic Dot1L inhibition

**Figure 5.**
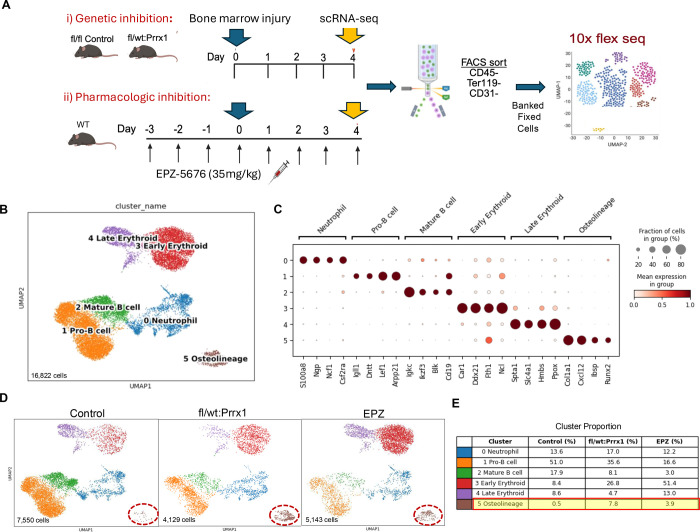
Expanded osteolineage cell cluster under conditions of either Dot1L genetic depletion or pharmacologic inhibition following bone marrow injury in mice. **(A**) Schematic overview of mechanical bone marrow injury model and scRNA-seq workflow. Genetic perturbation approach compared Dot1L^fl/fl^ and Dot1L^fl/wt^:Prrx1 mice. For pharmacologic inhibition, WT C57BL6 mice were treated twice daily with 35mg/kg EPZ-5676 (i.p., 3-day pretreatment, and 4 days post-injury). Mice were harvested 4 days post-injury. Lineage depleted (CD45^−^Ter119^−^CD31^−^) bone marrow cells were purified and pooled from n= xx mice per condition, then processed for 10x Genomics Flex scRNA-seq. (**B**) UMAP visualization of 16,822 cells following unbiased clustering and annotation based on canonical marker genes. (**C**) Dot plot showing top discriminative marker gene sets for each cell cluster, compared to cells from every other cluster. Dot size denotes fraction of cells expressing each gene, and color scales indicate normalized gene expression. (**D**) UMAP projections of individual samples. The “osteolineage” cluster is circled. (**E**) Quantification of cell type proportions across clusters. The osteolineage population increased from 0.5% in control to 3.9% in EPZ-5676, and 7.8% in Dot1L^fl/wt^:Prrx1.

Based on our *in vitro* and *in vivo* observations, we reasoned that Dot1L normally maintains quiescence in bone marrow stromal progenitors and functions as an epigenetic brake to restrain osteogenic induction. To test this, we utilized an established mechanical bone marrow injury challenge that induces a rapid intramembranous bone formation response within the marrow cavity (Matsushita et al., 2020; Stetsiv et al., 2025b). For rigor, we employed complementary genetic and pharmacologic strategies to reduce Dot1L activity (Figure 5A). Genetic haploinsufficiency in the Prrx1 lineage (Dot1L^fl/wt^:Prrx1Cre) provided a stable, lineage-restricted reduction of Dot1L dosage within skeletal stromal progenitors. Treatment with EPZ-5676, (a highly selective, S-adenosyl-methionine (SAM) competitive inhibitor of Dot1L histone methyltransferase activity with >37,000-fold selectivity over other methyltransferases) (Daigle et al., 2013; Godfrey et al., 2019), allowed temporally controlled, acute suppression of Dot1L catalytic activity without eliciting detrimental effects on skeletal development. Efficacy of the EPZ-5676 dosing in mice was established, as shown by loss of H3K79me2 protein expression in bone marrow from treated animals (Supplemental Figure 3).

Single-cell RNA-seq was performed on lineage-depleted marrow cells isolated 4 days after mechanical injury, a time point chosen to capture stromal activation and lineage priming before overt mineralized bone formation. To rigorously interrogate Dot1L-dependent injury-response programs and to distinguish effects intrinsic to skeletal progenitors from those arising through acute enzymatic inhibition, we isolated Lineage^−^ cells from injured control, Dot1L^fl/wt^:Prrx1, and EPZ-5676 treated mice and processed the samples using the 10x Genomics Flex workflow (Figure 5A). Flow cytometric enrichment of Lineage^−^ cells was comparable across samples (Supplemental Figure 4), and downstream preprocessing showed similar distributions of detected genes and total UMI counts across conditions before Harmony-based integration and batch correction (Supplemental Figure 5).

Integrated analysis and unsupervised clustering of 16,822 cells identified six transcriptionally distinct clusters representing the major stromal and hematopoietic populations present during early injury response (Figure 5B). Cluster identities were assigned based on canonical marker expression and top abundant genes in each cluster (Figure 5C). Cluster 0 corresponded to neutrophils and was characterized by high expression of *S100a8, Ngp, Ncf1, Csf2ra*. Cluster 1 consisted of Pro-B cells expressing *Igll1, Dntt, Lef1*, and *Arpp21*, while Cluster 2 represented mature B cells characterized by *Igkc, Ikzf3, Blk,* and *Cd19*. Clusters 3 and 4 corresponded to early and late erythroid populations, respectively with Cluster 3 marked by *Car1, C1qtnf12, Gnl3*, and *Ddx21* and Cluster 4 enriched of late erythroid cells enriched for *Alas2, Ank1, Spta1, Slc25a37*. Notably, Cluster 5 delineated a distinct osteolineage population characterized by the coordinated up-regulation of hallmark osteoblast transcription factors, and bone matrix genes including *Runx2, Ibsp* and *Col1a1*.

Comparison of UMAP projections revealed similar overall topologies among Dot1L^fl/wt^:Prrx1, EPZ-5676-treated, and control samples. However, there were distinct differences in the relative abundance of specific cell populations (Figure 5D). Quantification of cluster proportions demonstrated a reduction in lymphoid populations and a concomitant expansion of erythroid cells with reduced Dot1L activity (Figure 5E). Pro-B cells (Cluster 1) decreased from 51% in controls to 35.6% in Dot1^fl/wt^:Prrx1 mice and 16.6% following EPZ-5676 treatment, while mature B cells (Cluster 2) declined from 17.9% to 8.1% and 3%, respectively. In contrast, early erythroid progenitors (Cluster 3) expanded from 8.4% in controls to 26.8% in Dot1L haploinsufficient mice and 51.4% in EPZ-5676-treated mice. Strikingly, the dominant Dot1L-dependent effect was observed within the osteolineage compartment (Cluster 5), which expanded from a rare population in control marrow (0.5%) to a major cellular component under conditions of reduced Dot1L activity. Notably, the frequency of cells within Cluster 5 was elevated in both the Dot1L^fl/wt^:Prrx1Cre sample and EPZ-5676-treated sample. This expansion was most pronounced in Dot1L^fl/wt^:Prrx1 mice (7.8%) and was also robustly recapitulated by acute *in vivo* pharmacologic inhibition with EPZ-5676 (3.9%). The shared and disproportionate amplification of osteolineage cells across both perturbation strategies underscores Dot1L as a central epigenetic constraint on early osteogenic activation during post-injury marrow regeneration.

### Sub-clustering of the osteolineage compartment showed expansion of Fibro-MSC and Cxcl12+ CAR cell populations after Dot1L perturbation.

**Figure 6.**
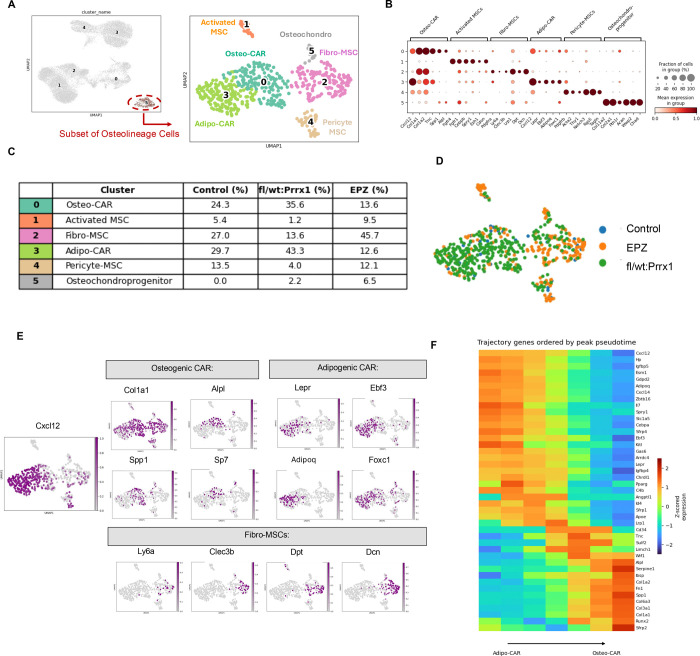
Transcriptional heterogeneity and context-dependent expansion of CAR and Fibro-MSC stromal states following Dot1L perturbation in injured bone marrow. (**A, B**) UMAP visualization of the osteolineage cluster that was extracted from the full dataset for secondary clustering. Dot plot compiling representative marker genes to identify cluster-specific sub-types. Dot size indicates the fraction of cells expressing each gene, and color intensity represents scaled mean expression. Sub-populations included Osteo-CAR (cluster 0), Activated MSCs (cluster 1); Fibro-MSCs (cluster 2); Adipo-CAR (cluster 3), Pericyte-MSCs (cluster 4) and Osteo-chondro progenitors (cluster 5). (**C**) Frequency of cells within each cluster across all samples. (**D**) UMAP projection shows distribution of each respective sample across predicted Adipo-CAR, Osteo-CAR and Fibro-MSC clusters. **(E)** Expression patterns of selected genes across CAR associated stromal populations across transcriptionally distinct CAR subpopulations illustrating coordinated variation in adipogenic associated genes (*Adipoq, Foxc1*), core CAR identity markers (*Cxcl12, Lepr, Ebf3*), and osteogenic and extracellular matrix–associated genes (*Col1a1, Alpl, Spp1, Sp7*).**(B)** Heat map showing pseudotime ordered gene expression dynamics across CAR associated stromal cells, with genes arranged by peak expression along the adipogenic to osteogenic biased CAR continuum, highlighting structured transcriptional state variation within the CAR compartment under conditions of reduced Dot1L activity.

To more precisely define the stromal progenitor subsets expanded by reduced Dot1L activity during early injury repair, we performed high-resolution sub-clustering of Cluster 5, the osteolineage enriched group identified in our initial UMAP analysis (Figure 6A). Given that the global Lineage^−^ compartment comprises a broad and heterogenous spectrum of skeletal progenitor states, this approach enabled a refined resolution of SSPC subsets selectively responsive to Dot1L perturbation. Sub-clustering resolved six transcriptionally distinct stromal subpopulations, including two subsets of Cxcl12-abundant reticular cells (CAR; clusters 0 and 3), activated MSCs (cluster 1), fibroblast-like mesenchymal stromal cells (Fibro-MSCs; cluster 2), pericyte-like MSCs (Cluster 4) and osteochondro-progenitors (Cluster 5). Dot plot analysis confirmed the expected distribution of canonical marker genes across these populations (Figure 6B). Consistent with the nomenclature for Cxcl12-abundant reticular subsets, the Osteo-CAR cluster (Cluster 0) was enriched for *Cxcl12, Lepr, Ebf3, Adipoq,* and *Foxc1*, whereas Fibro-MSCs expressed genes such as *Dcn, Dpt, Clec3b,* and *Ly6a* (Figure 6E), consistent with established marrow stromal identities (Jeffery et al., 2022; Mo et al., 2022; Zhou et al., 2014b; Zou et al., 2025).

Quantitative comparison of sub-cluster proportions across experimental conditions demonstrated pronounced and selective expansion of distinct osteolineage populations following Dot1L perturbation with distinct patterns emerging between genetic and pharmacologic approaches (Figure 6C). Genetic Dot1L haploinsufficiency in the Prrx1 lineage (Dot1^fl/wt^:Prrx1Cre) resulted in preferential expansion of the two CAR sub-sets (CXCL12HI / Osteo-CAR and CXCL12LO / Adipo-CAR). Specifically, Osteo-CAR cells increased from 24.3% in control marrow to 35.6% in Dot1l^fl/wt^;Prrx1Cre mice, while Adipo-CAR cells expanded from 29.7% to 43.3%.

In contrast, acute *in vivo* pharmacologic inhibition with EPZ-5676 produced a distinct response marked by preferential enrichment of Fibro-MSCs population. Fibro-MSCs increased from 27% of osteolineage cells in control samples to 45.7% following EPZ-5676 treatment, while representing only 13.6% of osteolineage cells in Dot1^fl/wt^:Prrx1Cre mice. Notably, both CXCL12-HI and CXCL12-LO CAR populations were reduced in EPZ-5676-treated samples (to 12.6% and 13.6%, respectively), highlighting a context-dependent effect of genetic versus pharmacologic Dot1L reduction. UMAP projection of sample contributions across the osteolineage manifold further corroborated these findings, revealing dominant enrichment of the Dot1^fl/wt^:Prrx1 cells within CAR associated clusters and preferential localization of EPZ-5676-treated cells within the Fibro-MSC compartment (Figure 6D).

To further validate the identity and functional characteristics of stromal populations expanded following Dot1L perturbation, we examined the expression patterns of canonical marker genes across osteolineage subclusters. Feature plot analyses (Figure 6E) confirmed that CAR subpopulations retained high expression of core niche-associated genes, including *Cxcl12, Lepr,* and *Ebf3*, consistent with established Cxcl12-abundant reticular cell identity. Within this compartment, Osteo-CAR cells showed enrichment of osteogenic-associated genes such as *Col1a1, Alpl,* and *Sp7*, whereas Adipo-CAR cells expressed adipogenic and niche-supportive markers including *Adipoq* and *Foxc1*. In contrast, the Fibro-MSC population was distinguished by elevated expression of extracellular matrix and fibroblast-associated genes, including *Dcn, Dpt, Clec3b,* and *Ly6a* (Figure 6E–F), consistent with previous descriptions of fibroblast-like stromal states in the bone marrow. Comparative visualization of these marker signatures (Figure 6F) highlights clear transcriptional distinctions between CAR-associated and Fibro-MSC populations, supporting their classification as discrete stromal progenitor states.

Together, these marker expression patterns confirm that Dot1L perturbation alters the relative abundance of well-defined stromal populations, rather than inducing nonspecific transcriptional changes, and reinforce the conclusion that genetic and pharmacologic reductions of Dot1L activity differentially bias the composition of the osteolineage compartment during early marrow repair.

### Dot1L haploinsufficiency enhances bone formation following mechanical bone marrow injury.

**Figure 7.**
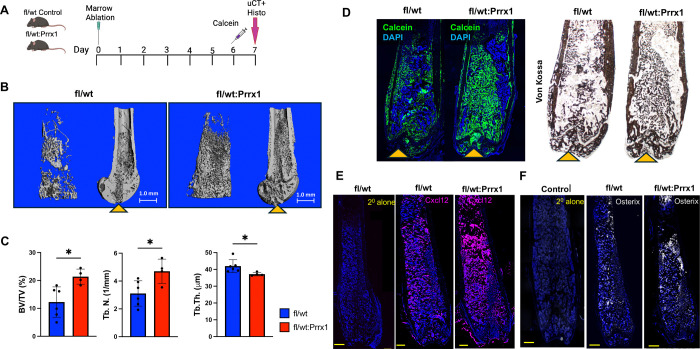
Dot1L haploinsufficiency within the Prrx1 lineage accelerates injury-induced bone regeneration *in vivo*. (**A**) Schematic for bone marrow injury models and phenotypic analysis of new bone formation. Mechanical bone marrow injury was induced in the left femur of skeletally mature Dot1L^fl/fl^ or Dot1L^fl/wt^:Prrx1Cre mice (day 0). Calcein mineral label was delivered by i.p. injection at 20 mg/kg on days 5 and 6 post-surgery, and tissue harvested on day 7. **(B)** Representative micro-CT images of trabecular cores and femur cross sections showing the path of the needle injury (yellow arrowhead) in Dot1L^fl/wt^ controls and Dot1L^fl/wt^:Prrx1 female mice on day 7 post-injury. (**C**) Quantitative trabecular bone analysis showing a significant increase in bone volume fraction (BV/TV) and trabecular number (Tb. N.), as well as a significant decrease in trabecular thickness (Tb. Th) in the regenerated bone of the Dot1L^fl/wt^:Prrx1 mice compared to controls. Graphs presented as mean ± SD; * *p* ≤ 0.05. (**D**) Representative images of Calcein mineral label (green) showing an increase in fluorescently labeled tissue in the Dot1L^fl/wt^:Prrx1 mice. Subsequent sections showing an increase in mineral deposition by Von Kossa stain with Dot1L partial deletion compared to Dot1L^fl/wt^ control mice. Yellow arrowheads indicate path of injury**. (E, F)** Representative immunofluorescence images of injured femoral marrow sections from Dot1L^fl/wt^ control and Dot1L^fl/wt^:Prrx mice at day 7 post-injury, showing enhanced Cxcl12 staining and an increased abundance of Osterix (Osx)–positive osteogenic progenitors within the regenerated marrow is observed in Dot1L^fl/wt^:Prrx mice compared with controls. mages shown are representative sections from 3–4 independent mice per genotype. Secondary-antibody–only controls were included for both CXCL12 and Osterix immunostaining to confirm signal specificity.

To determine whether Dot1L haploinsufficiency within the Prrx1 lineage enhances bone regeneration, we analyzed trabecular bone parameters in the contralateral uninjured) and injured femurs from female Dot1L^fl/wt^:Prrx1Cre mice and Dot1L^fl/fl^ control littermates seven days after mechanical bone marrow injury (Figure 7A). In non-injured femurs, microCT analysis demonstrated reduced trabecular bone in Dot1L^fl/wt^:Prrx1Cre mice versus Dot1L^fl/wt^ controls (Supplemental Figure 6 A-B), consistent with our published data showing developmental defects in forming the cartilage template needed for endochondral-mediated bone formation (Sutter et al., 2021). In contrast, quantitative microCT analyses revealed robust and reproducible increases in bone volume fraction (BV/TV) and trabecular number (Tb.N.), accompanied by a concomitant reduction in trabecular thickness (Tb.Th.), in injured femurs from Dot1L^fl/wt^:Prrx1 Cre mice compared with controls (Figure 7B–C). Specifically, mean BV/TV increased from 12.3% (± SD) in control mice to 21.4% (± SD) in Dot1L^fl/wt^:Prrx1Cre mice, representing a near two-fold enhancement in trabecular bone volume. Trabecular number increased from 3.1 mm^−1^ in controls to 4.7 mm^−1^ in Dot1L haploinsufficient mice. In contrast, trabecular thickness was significantly reduced in Dot1L^fl/wt^:Prrx1Cre femurs (37 μm ± SD) relative to controls (42 μm ± SD). The combination of increased trabecular bone volume and trabecular number with reduced trabecular thickness is characteristic of newly formed woven bone and is consistent with accelerated regenerative bone formation following Dot1L dosage reduction.

Dynamic calcein labeling, further revealed markedly increased mineralizing surface area within the injury site of Dot1L haploinsufficient mice, indicative of heightened osteogenic activity and accelerated new bone deposition (Figure 7D). Consistent with these findings, von Kossa staining of adjacent sections showed increased mineral deposition within the injury site in Dot1L haploinsufficient marrow (Figure 7D). Importantly, these structural and histomorphometric changes were accompanied by pronounced cellular remodeling of the injury niche. Injured femurs from Dot1L haploinsufficient mice exhibited increased Cxcl12 immunoreactivity within the regenerated marrow space, consistent with enhanced presence or persistence of CXCL12^+^ stromal populations during repair (Figure 7E). In parallel, Osterix (Sp7) staining revealed a substantial increase in osteoprogenitor cells localized to the injury site (Figure 7F), directly linking reduced Dot1L dosage to augmented osteolineage engagement *in vivo*. Together, these integrated analyses demonstrate that Dot1L haploinsufficiency potently amplifies the early regenerative response to marrow injury, coupling expansion of injury-responsive stromal and osteogenic progenitor populations with accelerated bone formation and mineralization.

## DISCUSSION

Skeletal regeneration in adult bones requires rapid activation and coordination of stromal progenitor populations that are otherwise maintained in a quiescent state. While extensive work has defined transcriptional programs and signaling pathways governing this response, far less is known about the epigenetic mechanisms that regulate the readiness of adult skeletal progenitors to respond to injury. Here, we identify the H3K79 methyltransferase Dot1L as a key epigenetic regulator that limits early progenitor activation in the adult bone marrow and constrains the magnitude of the regenerative response. Analysis of published scRNAseq datasets from murine bone marrow shows that Dot1L is broadly expressed across skeletal stromal and progenitor cell populations enriched for *Cxcl12*, and *Lepr* (Matsushita et al., 2020), positioning Dot1L to regulate stromal progenitor activation and fate transitions during skeletal regeneration. Functionally consistent with this expression profile, partial reduction of Dot1L activity through genetic haploinsufficiency in the Prrx1 lineage (Dot1L^fl/wt^:Prrx1-Cre) enhanced regenerative bone formation *in vivo*, reframing Dot1L as an epigenetic brake on progenitor activation. In parallel, acute pharmacologic inhibition of Dot1L using EPZ-5676 provided a complementary approach for interrogating early transcriptional and cellular responses by scRNA sequencing.

Within this framework, our findings integrate with emerging models of skeletal stem and progenitor cell behavior during injury repair. Prior work demonstrates that *Cxcl12-*expressing stromal cells undergo rapid injury-induced- identity transitions characterized by loss of niche factor expression, proliferation, migration, and activation of osteogenic transcriptional programs (Matsushita et al., 2020). Our data suggest that Dot1L acts upstream of this transition by maintaining chromatin states that stabilize progenitor quiescence and stromal identity under homeostatic conditions. By setting this activation threshold, Dot1L shapes the size and composition of stromal populations recruited into the regenerative niche during repair.

To determine whether the enhanced activation and expansion of stromal progenitors observed *in vivo* reflects altered intrinsic lineage competence, we next considered the differentiation capacity of Dot1L-deficient bone marrow stromal cells under defined *in vitro* conditions. Our *in vitro* assays demonstrate that Dot1L deficiency enhances both osteogenic and adipogenic differentiation capacity of adult skeletal progenitors, alongside increased clonogenicity and proliferative output. These findings align with Dot1L’s established role in regulating transcriptional programs that maintain cellular identity and proliferation (Du et al., 2025; Roidl et al., 2016; Wu et al., 2024). Prior studies in chondrocytes and limb bud mesenchyme further show that Dot1L loss accelerates differentiation trajectories and disrupts the balance between proliferation and maturation (Sutter et al., 2021). In this context, the enhanced ALP^+^ colony formation and increased adipogenic differentiation observed here support a model in which Dot1L functions as an epigenetic “gatekeeper” that regulates the timing and permissiveness of mesenchymal lineage progression, rather than enforcing commitment to a single lineage outcome.

Skeletal regeneration in adult bone requires rapid activation and coordination of stromal progenitor populations that are otherwise maintained in a quiescent state. Here, we identify Dot1L as a central epigenetic regulator that constrains this early regenerative response in the adult bone marrow. Partial reduction of Dot1L activity within the Prrx1 lineage markedly amplified marrow repair following injury, coupling expansion of injury-responsive stromal populations with accelerated bone formation.

At the cellular level, single cell transcriptomic analyses further demonstrate- that Dot1L functions as a central epigenetic regulator of adult bone marrow stromal progenitor activation during marrow repair. Across injury conditions, Dot1L reduction resulted in marked remodeling of osteolineage and osteo-adipo progenitor compartments with the specific stromal populations expanded depending on the mode and timing of Dot1L perturbation. Multiple populations including Cxcl12^+^ CAR cells (Liao et al., 2025; Matsushita et al., 2020), Lepr^+^ stromal progenitors (Zhou et al., 2014a), Adipoq^+^ marrow progenitors (Liao et al., 2025), and Ebf3-expressing niche supportive cells (Galan-Diez and Kousteni, 2018; Seike et al., 2018) were expanded, indicating a shared Dot1L-dependent regulatory program that maintains stromal progenitor quiescence under homeostatic conditions. These stromal states closely recapitulate marrow niche heterogeneity described in prior single cell- studies, particularly Cxcl12^+^ CAR cells that support hematopoiesis and contribute to osteoblast formation during injury repair (Matsushita et al., 2020). CAR cells are among the earliest stromal populations to undergo injury-induced identity transitions, characterized by suppression of niche-supportive programs and activation of osteogenic transcriptional networks. Their preferential enrichment following Dot1L reduction implicates Dot1L as a key constraint on activation and expansion of these injury-responsive stromal progenitors. Importantly, this enhanced regenerative response was supported by concordant structural, cellular, and molecular readouts: Dot1L haploinsufficiency increased trabecular bone volume and mineralization, expanded CXCL12^+^ stromal cell presence within the regenerated marrow space, and robustly elevated Osterix^+^ osteoprogenitor abundance at the injury site. Together, these findings establish Dot1L as a key epigenetic brake that limits the magnitude of stromal progenitor recruitment and osteolineage engagement during early skeletal repair. Interestingly, these transcriptional changes were reinforced by tissue-level analyses, as Dot1L haploinsufficient mice exhibited increased CXCL12 immunoreactivity and elevated Osterix-positive osteoprogenitor abundance within the injury site, providing spatial and cellular validation of the injury-responsive progenitor expansion identified by single-cell profiling.

Notably, scRNA-seq revealed context-dependent effects of genetic versus pharmacologic Dot1L perturbation. Lineage-restricted Dot1L haploinsufficiency led to robust expansion of both CXCL12-HI (Adipo-CAR) and CXCL12-LO (Osteo-CAR) CAR populations, whereas acute enzymatic inhibition with EPZ-5676 was associated with relative depletion of CAR cells and enrichment of fibroblast-like stromal states. These divergent outcomes likely reflect differences in duration, timing, and cellular specificity of Dot1L suppression: sustained genetic reduction permits CAR accumulation during early repair, whereas acute inhibition may accelerate stromal state progression, resulting in transient depletion of identifiable CAR identities at the analyzed time point.

High-resolution sub-clustering revealed transcriptionally distinct Adipo-CAR and Osteo-CAR states within the broader CAR compartment. Adipo-CAR cells were enriched for adipogenic and niche-supportive programs, whereas Osteo-CAR cells preferentially expressed osteogenic and extracellular matrix genes and predominated during injury-induced bone formation (Jeffery et al., 2022; Matsushita et al., 2020). Dot1L haploinsufficiency biased CAR state occupancy toward osteogenic-primed CAR identities.

Viewed in the context of prior work, the stromal populations expanded following Dot1L reduction correspond to progenitor states previously shown to play central roles in skeletal regeneration. Prior studies have established that CAR-derived and related marrow stromal progenitors give rise to osteoblasts and coordinate marrow reorganization during repair, while distinct but overlapping progenitor subsets balance osteogenesis with metabolic and niche-supportive functions (Galan-Diez and Kousteni, 2018; Liao et al., 2025; Seike et al., 2018; Zhou et al., 2014a). Together, these data support a model in which Dot1L acts as an epigenetic brake on early stromal progenitor activation and CAR state progression, limiting the pool of osteolineage-primed progenitors available for regeneration.

A key conceptual advance of our study is the recognition that Dot1L functions as a highly context-dependent regulator of progenitor activation whose function shifts with developmental and tissue state. While Dot1L is broadly required during embryogenesis to support progenitor proliferation and lineage progression across multiple organ systems (Feng et al., 2010; Ferrari et al., 2020; Gray de Cristoforis et al., 2020; Pursani et al., 2018; Roidl et al., 2016), accumulating evidence in adult tissues (Appiah et al., 2023; Aslam et al., 2021; Scheer et al., 2020) indicates a contrasting role in maintaining cellular identity and limiting lineage plasticity. Our findings extend this emerging paradigm to the adult bone marrow, where reduced Dot1L activity selectively amplifies injury-responsive stromal progenitor populations and accelerates their early regenerative engagement. Together, these data support a model in which Dot1L promotes lineage progression during development but in adult tissues, functions primarily to restrain the timing and magnitude of progenitor activation during adult tissue repair.

Despite these insights, few limitations of the current study should be noted. Use of the Prrx1Cre driver targets a broad limb mesenchyme-derived lineage, limiting definitive assignment of effects to specific progenitor subsets. Future studies will use inducible strategies for genetic depletion of Dot1L in adult stromal progenitors, avoiding any developmental changes. scRNA-seq was performed only in the injured state, leaving unresolved whether Dot1L loss alters progenitor priming under homeostatic conditions. In addition, transcriptomic profiling was conducted using probe-based scRNA seq of fixed cells, which does not provide transcriptome-wide coverage or capture low abundance transcripts and dynamic transcriptional processes. Dot1L may also exert H3K79-independent functions (Cao et al., 2020), thus direct chromatin profiling will be required to distinguish methylation-dependent mechanisms. Finally, pooling strategies limited animal-level replication, although the magnitude and consistency of effects support biological relevance.

In summary, these studies identify Dot1L as a previously unrecognized epigenetic regulator restraining activation, proliferation, and lineage priming of adult skeletal stromal and progenitor cells. Reducing Dot1L dosage in *Prrx1*^+^ progenitors expand the injury responsive SSPC compartment, promotes early osteoprogenitor emergence, and enhances regenerative bone formation *in vivo*. By relieving a shared epigenetic constraint imposed by H3K79 methylation across multiple progenitor states, Dot1L haploinsufficiency amplifies the pool of osteolineage-competent cells available for repair, establishing a foundation for future studies exploring temporal modulation of Dot1L to enhance endogenous bone regeneration.

## Supplementary Material

Supplement 1

## Figures and Tables

**Table 1.** qPCR Primers

Gene	Forward (5’ -> 3’)	Reverse (5’ -> 3’)
*B-actin*	AGA TGT GGA TCA GCA AGC AG	GCG CAA GTT AGG TTT TGT CA
*Alp*	TGA CCT TCT CTC CTC CAT CC	CTT CCT GGG AGT CTC ATC CT
*Sp7/Osx*	GAG GAG TCC ATT GGT GCT TGA GA	GGA TGG CGT CCT CTC TGC TTG AG
*Ibsp*	CGC CAC ACT TTC CAC ACT CTC	CTT CCT CGT CGC TTT CCT TCA
*Ocn*	TGC TTG TGA CGA GCT ATC AG	GAG GAC AGG GAG GAT CAA GT

**Table 2.** Antibodies

Antibody	Application	Fluorophore	Vendor	Catalog #
Anti-CD45	Flow	FITC	Biolegend	103107
Anti-CD31	Flow	FITC	Biolegend	102405
Anti-Ter119	Flow	FITC	Biolegend	116205
Anti-CD45 (Clone 30-F11)	FACS/Flow	Pacific Blue	Biolegend	103125
Anti-CD31 (Clone 390)	FACS/Flow	Pacific Blue	Biolegend	102421
Anti-Ter119	FACS/Flow	APC	Biolegend	116211
Anti-H3K79me2	WB	-	Abcam	3594
Anti-b-actin	WB	-	Cell Signaling Tech	4970
Hoechst 33342	Flow	-	Thermo Fisher	C10337-G
Zombie Live/Dead	Flow	NIR	Biolegend	423105
UltraComp Compensation Beads	Flow	-	Thermo Fisher	01-3333-41
Rb Polyclonal anti-Cxcl12	IF	-	Thermofisher	PA5-89116
Anti-Sp7/Osterix	IF	-	Abcam	Ab209484
AF647 anti-Rabbit	IF	AF647	Thermofisher	A21244
