# Supplementary material for: Inhibition of Dot1L Histone Methyltransferase Expands Bone Injury-Responsive CXCL12+ Stromal Progenitors
Source: bioRxiv. 2026 Apr 9:2026.04.06.716818. Preprint. [Version 1] doi: 10.64898/2026.04.06.716818 (PMC13082038; doi:10.64898/2026.04.06.716818)
Supplement: Supplement 1 [file NIHPP2026.04.06.716818v1-supplement-1.pdf]

Supplemental Figure 1

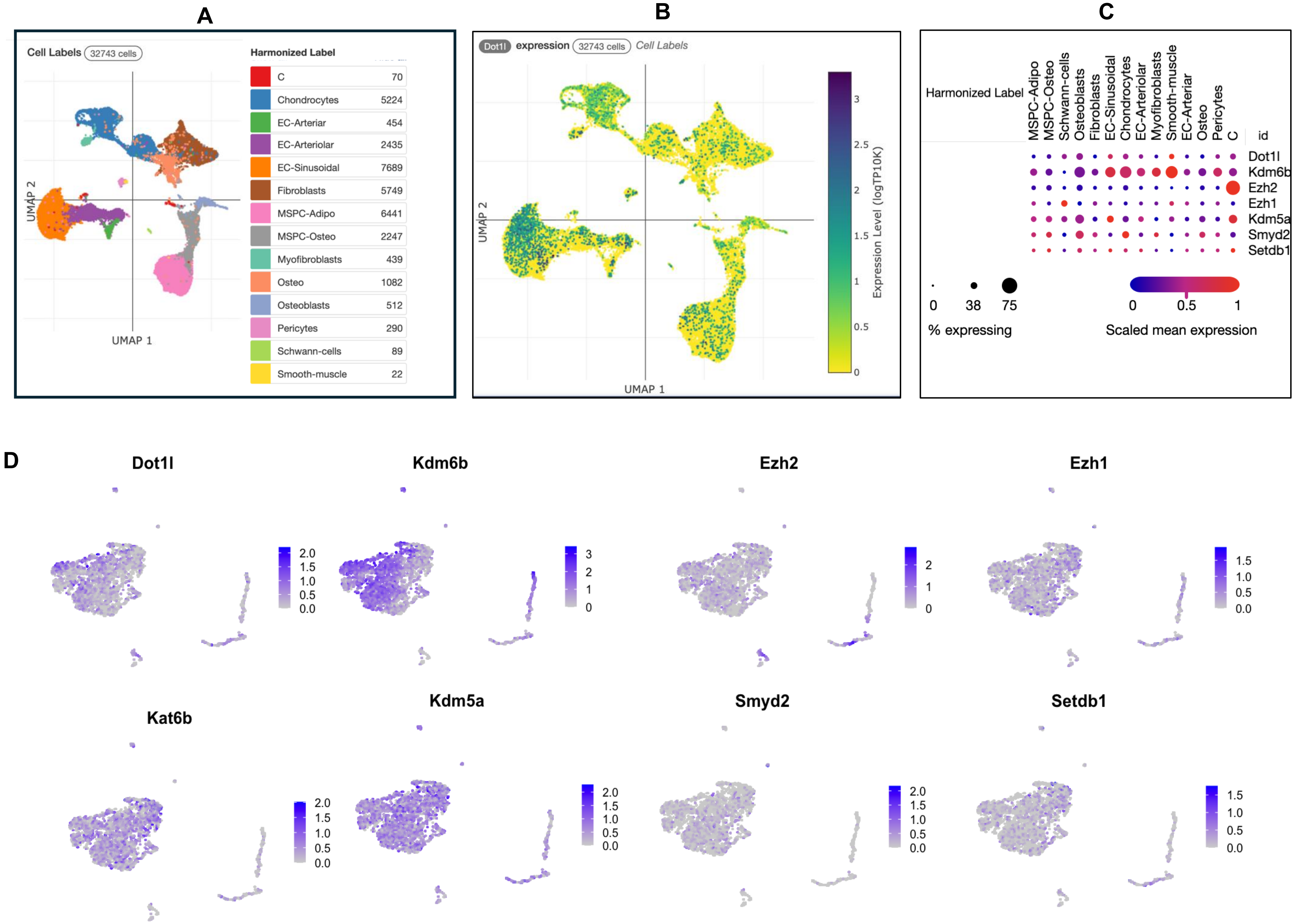

bioRxiv preprint doi: https://doi.org/10.64898/2026.04.06.716818; this version posted April 2026. The copyright holder for this preprint (which was not certified by peer review) is the author/funder, who has granted bioRxiv a license to display the preprint in perpetuity. It is made available under aCC-BY 4.0 International license.

Supplemental Figure 2

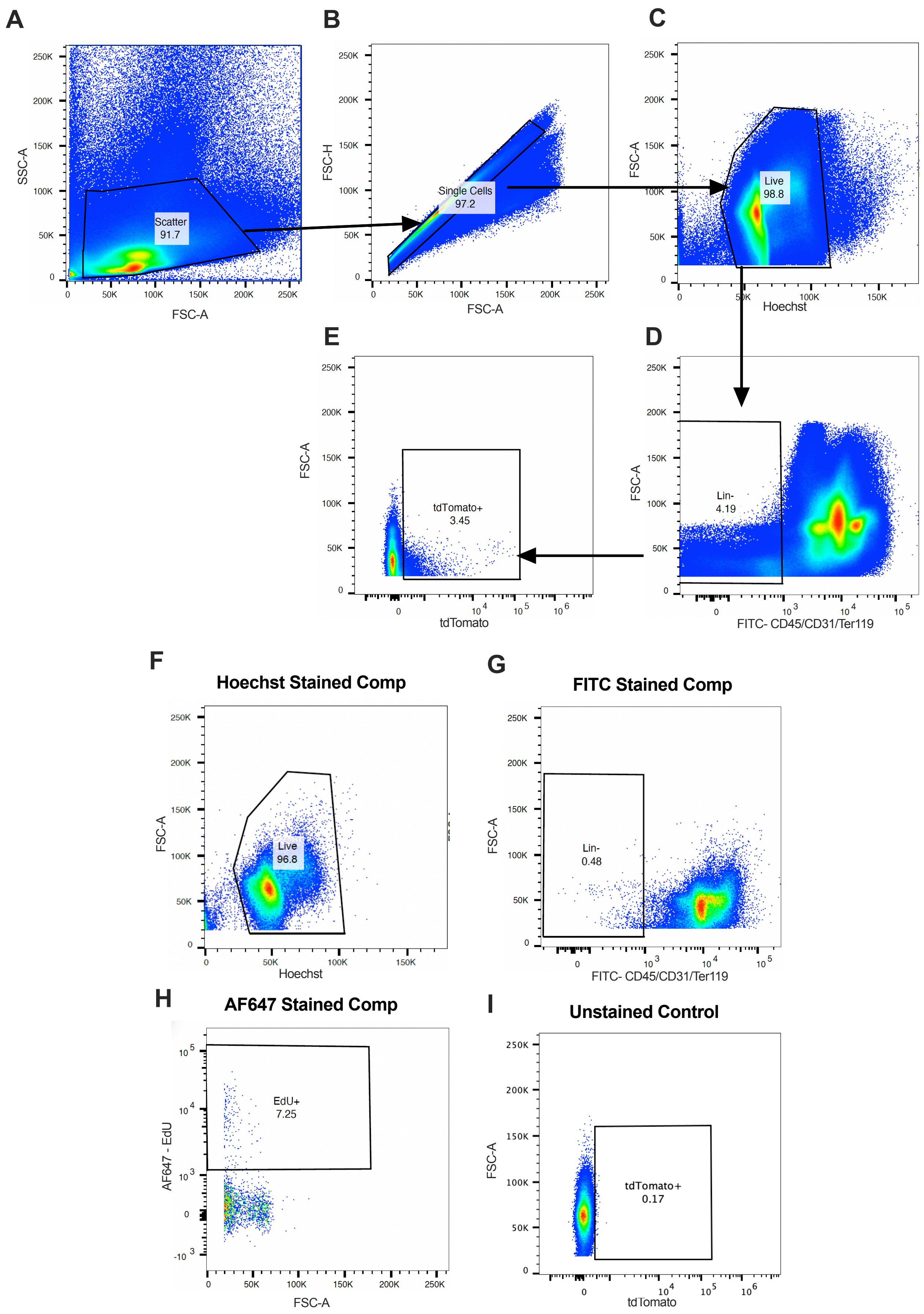

bioRxiv preprint doi: https://doi.org/10.64898/2026.04.06.716818; this version posted April 9, 2026. The copyright holder for this preprint (which was not certified by peer review) is the author/funder, who has granted bioRxiv a license to display the preprint in perpetuity. It is made available under aCC-BY 4.0 International license.

Supplemental Figure 3

A

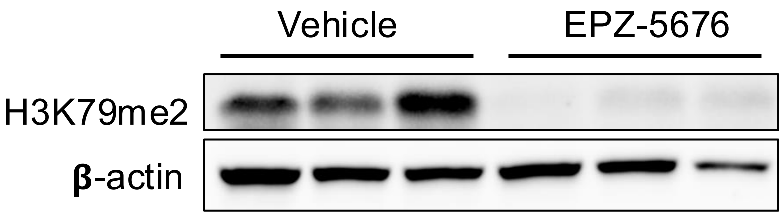

B

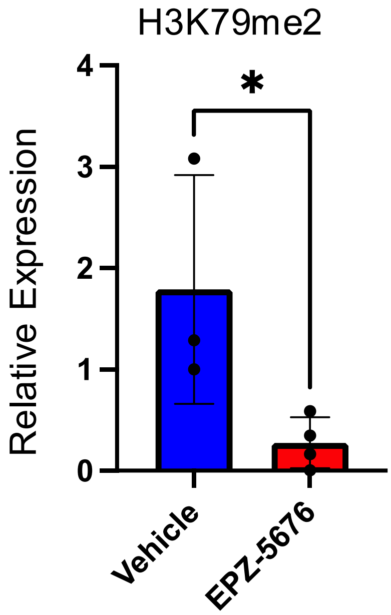

Supplemental Figure 4

bioRxiv preprint doi: https://doi.org/10.64898/2026.04.06.716818; this version posted April 9, 2026. The copyright holder for this preprint (which was not certified by peer review) is the author/funder, who has granted bioRxiv a license to display the preprint in perpetuity. It is made available under aCC-BY 4.0 International license.

Control

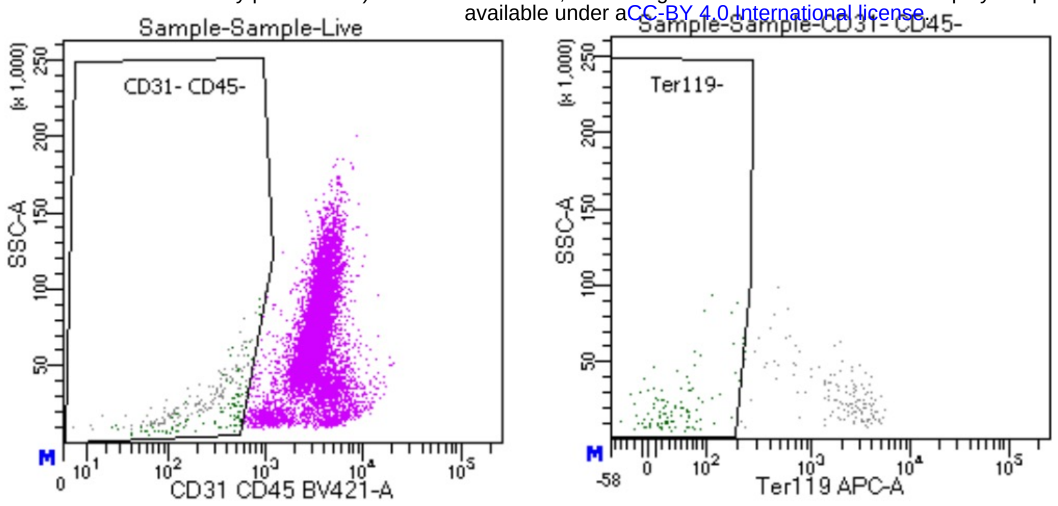

fl/wt:Prrx1

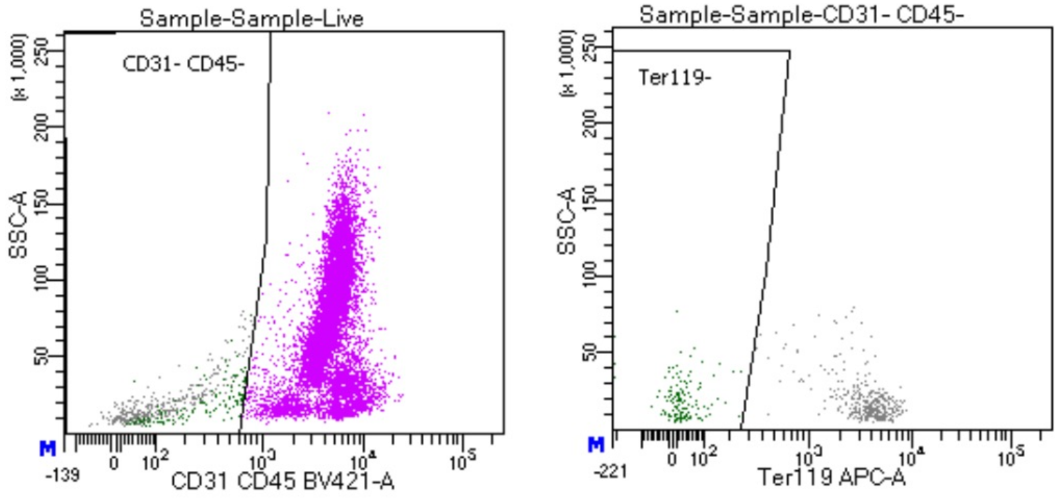

EPZ Treated

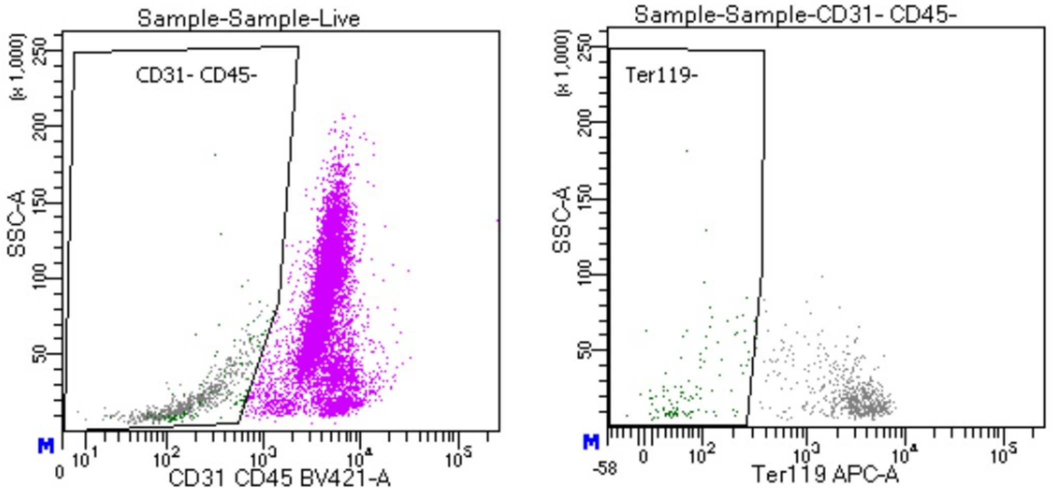

Sample	CD45-CD31- % of parental (single live)	Ter119- % of parental (single live CD45- CD31-)	% CD45-CD31- Ter119- in single live population
Control	2.5%	42.2%	1.0%
fl/wt:Prrx1	5.0%	26.7%	1.3%
EPZ	6.8%	15.8%	1.1%

Supplemental Figure 5

A

Pre-Filtering

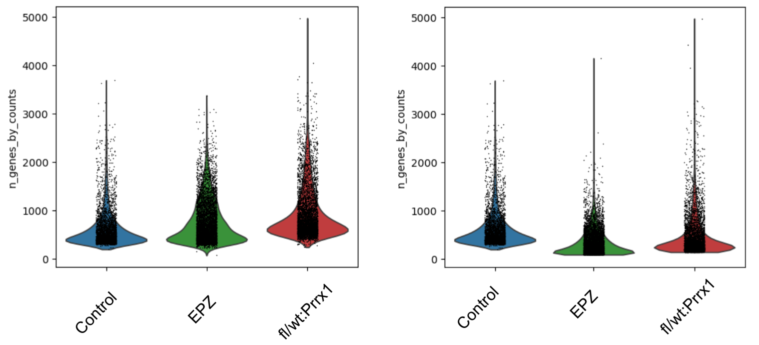

B

Post-Filtering

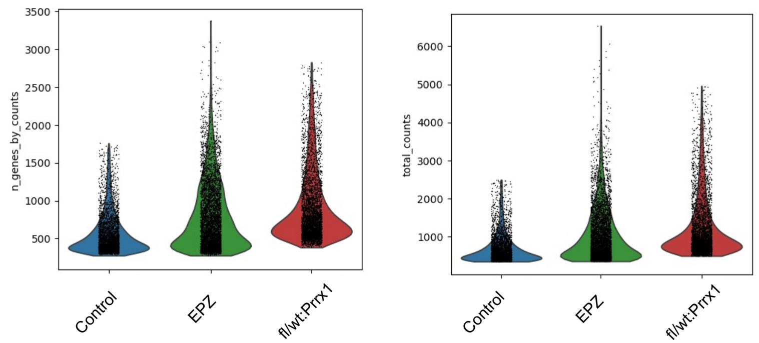

C

Filter Applied	Package or Thresholds Used to Filter Out Low Quality Cells
**Mitochondrial, Ribosomal, & Hemoglobi n Genes**	>5 median absolution deviations
**Gene counts, Total counts, Proportion of top 20 expressed genes**	>5 median absolution deviations
**Genes**	Min 200 genes expressed, min 10 cells expressing
**Batch**	HarmonyPy (v0.0.10)
**Ambient RNA**	SoupX (v1.6.2)
**Doublets**	scDblFinder (v1.23.4)

bioRxiv preprint doi: https://doi.org/10.64898/2026.04.06.716818; this version posted April 9, 2026. The copyright holder for this preprint (which was not certified by peer review) is the author/funder, who has granted bioRxiv a license to display the preprint in perpetuity. It is made available under aCC-BY 4.0 International license.

Supplemental Figure 6

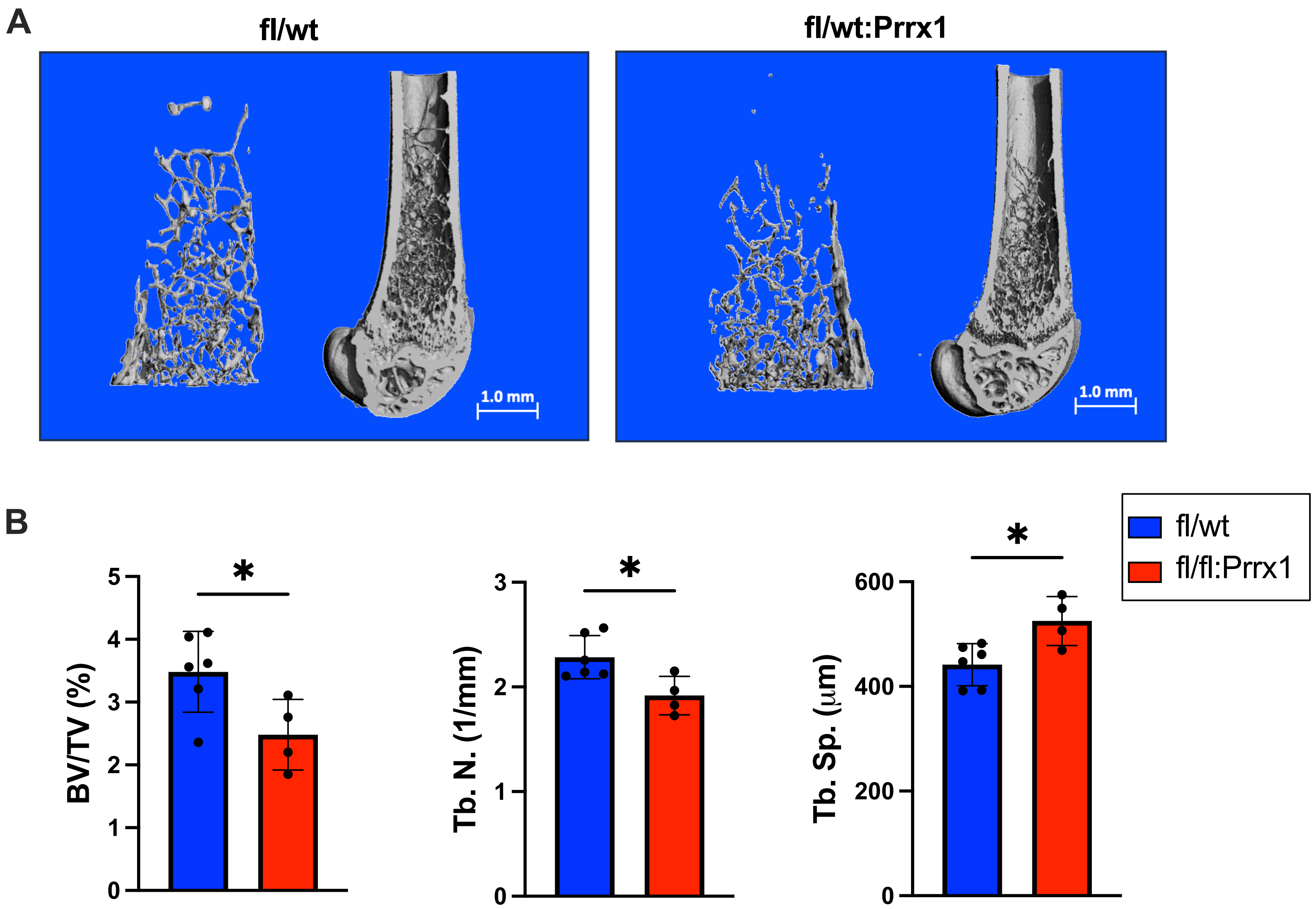

bioRxiv preprint doi: https://doi.org/10.64898/2026.04.06.716818; this version posted April 9, 2026. The copyright holder for this preprint (which was not certified by peer review) is the author/funder, who has granted bioRxiv a license to display the preprint in perpetuity. It is made available under aCC-BY 4.0 International license.

Supplemental Figure 1. (A) Integrated single-cell RNA-sequencing analysis of murine bone marrow cells compiled across multiple experimental and physiological conditions (Dolgalev and Tikhonova, 2021; Broad Single Cell Portal, study SCP1248). UMAP representation of transcriptionally defined clusters annotated based on established lineage and niche marker expression, including stromal, hematopoietic, endothelial, and other niche-associated populations. **(B)** Feature plot showing Dot1L expression projected onto the clustered dataset, demonstrating broad transcript detection across marrow populations with consistent expression within multiple stromal clusters. (**C)** Dot plot summarizing scaled expression levels (color scale) and the fraction of cells expressing Dot1L alongside selected histone-modifying enzymes and chromatin-associated regulators across stromal clusters, situating Dot1L within a broader epigenetic regulatory landscape active in bone marrow stromal cells. **(D)** Expression of epigenetic regulators across Cxcl12^{+} bone marrow niches. UMAP feature plots showing expression patterns of selected histone modifiers associated with regulation of osteogenic differentiation regulation across single-cell transcriptomes from fluorescence-activated cell sorted Cxcl12^{+} bone marrow stromal cells (GSE136979; Matsushita *et al.*, 2020).

Supplemental Figure 2. Flow cytometry gating strategy and controls for *in vivo* EdU proliferation analysis. Sequential gating strategy used to identify Lineage^{-} (CD45^{-}CD31^{-}Ter119^{-}) bone marrow stromal cells and Prrx1-lineage tdTomato^{+} cells for EdU proliferation analysis. **(A)** Debris were excluded by first gating on forward scatter (FSC-A) versus side scatter (SSC-A). **(B)** Singlets were isolated by gating on FSC-H versus FSC-A, **(C)** followed by selection of nucleated cells based on Hoechst fluorescence. **(D)** Lineage-negative stromal cells were identified by excluding FITC^{+} CD45^{+}, CD31^{+}, and Ter119^{+} hematopoietic and endothelial populations. **(E)** Within the Lineage^{-} compartment, tdTomato fluorescence was used to identify Prrx1-Cre labeled mesenchymal lineage cells. **(F)** A Hoechst only stained compensation control and FMO-Hoechst (not shown) were used to establish the viability gate. **(G)** A FITC-only stained compensation control (CD45/CD31/Ter119 panel) and FMO-FITC (not shown) were used to define the Lineage^{-} gate. **(H)** AF647-only stained compensation control, FMO-AF647 and unstained samples (not shown) were used to establish the EdU^{+} gate. **(I)** An unstained control from a WT mouse was included to define background fluorescence for tdTomato and all other detectors. All gating thresholds were determined using compensation and FMO controls to ensure accurate identification of Lineage^{-}, tdTomato^{+}, and EdU^{+} populations.

bioRxiv preprint doi: https://doi.org/10.64898/2026.04.06.716818; this version posted April 9, 2026. The copyright holder for this preprint (which was not certified by peer review) is the author/funder, who has granted bioRxiv a license to display the preprint in perpetuity. It is made available under aCC-BY 4.0 International license.

Supplemental Figure 3. Validation of dosing and efficacy of Dot1L inhibitor in vivo. (**A**) Representative western blot analyses of bone marrow cells from vehicle and EPZ-5676-treated mice. C57BL/6 mice were treated with the Dot1L inhibitor EPZ-5676 (35 mg/kg, twice daily) or vehicle (5% DMSO in corn oil) for 7 consecutive days. Bone marrow cells were harvested, subjected to red blood cell lysis, and analyzed by western blot for H3K79me2 protein expression. (**B**) Quantification of H3K79me2 signal normalized to β-actin. Data are presented as mean ± SD; * $p < 0.05$ by two-tailed unpaired t-test. n = 3 mice/group.

Supplemental Figure 4. FACS gating strategy and lineage-negative cell enrichment for single-cell RNA-seq. Representative flow cytometry plots showing sequential gating to enrich for Lineage^{-} stromal cells (CD45^{-}, CD31^{-}, Ter119^{-}) from Dot1L$^{fl/fl}$ control, Dot1L$^{fl/wt}$:Prrx1, and wild type EPZ-5676-treated samples used for single-cell RNA sequencing. Initial exclusion of hematopoietic (CD45^{+}) and endothelial (CD31^{+}) populations was followed by removal of erythroid lineage cells (Ter119^{+}). The accompanying table summarizes the percentage of cells within each gated population for each sample, demonstrating consistent enrichment of CD45^{-}CD31^{-}Ter119^{-} stromal cells across conditions.

Supplemental Figure 5. Quality control metrics and filtering criteria for single-cell RNA-seq analysis. (**A**) Violin plots showing the distribution of detected genes per cell and (**B**) total UMI counts per cell across Dot1L$^{fl/fl}$ control, Dot1L$^{fl/wt}$:Prrx1, and wild type EPZ-5676-treated samples following initial pre-processing. Overlaid points represent individual cells. **(C)** Table summarizing quality control filtering criteria applied prior to downstream analysis, including removal of cells with high mitochondrial (mt) or ribosomal/hemoglobin (Ribo, Hb) gene content (top 20%), gene inclusion thresholds (minimum 200 genes per cell and genes expressed in at least 10 cells), and batch correction using Harmony.

Supplemental Figure 6. microCT analysis of trabecular bone in contralateral (uninjured) femurs from 10-12 week old female mice. (**A**) Representative microCT images. (**B**) Quantitative trabecular bone analysis showed significantly reduced bone volume fraction (BV/TV) and trabecular number (Tb. N.), and concomitant increase in trabecular spacing (Tb. Sp.) in contralateral (non-injured) femurs from Dot1L$^{fl/wt}$:Prrx1 mice compared to controls. Graphs presented as mean ± SD; * $p \leq 0.05$.
